# Semi-supervised drug-protein interaction prediction from heterogeneous biological spaces

**DOI:** 10.1186/1752-0509-4-S2-S6

**Published:** 2010-09-13

**Authors:** Zheng Xia, Ling-Yun Wu, Xiaobo Zhou, Stephen TC Wong

**Affiliations:** 1 Bioinformatics and Bioengineering Program, The Methodist Hospital Research Institute, Weill Medical College, Cornell University, Houston, TX 77030, USA; 2 Institute of Applied Mathematics, Academy of Mathematics and Systems Science, Chinese Academy of Sciences, Beijing 100080, China

## Abstract

**Background:**

Predicting drug-protein interactions from heterogeneous biological data sources is a key step for *in silico *drug discovery. The difficulty of this prediction task lies in the rarity of known drug-protein interactions and myriad unknown interactions to be predicted. To meet this challenge, a manifold regularization semi-supervised learning method is presented to tackle this issue by using labeled and unlabeled information which often generates better results than using the labeled data alone. Furthermore, our semi-supervised learning method integrates known drug-protein interaction network information as well as chemical structure and genomic sequence data.

**Results:**

Using the proposed method, we predicted certain drug-protein interactions on the enzyme, ion channel, GPCRs, and nuclear receptor data sets. Some of them are confirmed by the latest publicly available drug targets databases such as KEGG.

**Conclusions:**

We report encouraging results of using our method for drug-protein interaction network reconstruction which may shed light on the molecular interaction inference and new uses of marketed drugs.

## Background

Developing a new drug is an expensive and time-consuming process that is subject to a variety of regulations such as drug toxicity monitoring and therapeutic efficacy. Meanwhile, there are thousands of FDA-approved drugs in the market and drugs in later phases of clinical trials. Finding the potential application in other therapeutic categories of those FDA-approved drugs by predicting their targets, known as drug repositioning, is an efficient and time-saving method in drug discovery [1]. Additionally, predicting interactions between drugs and target proteins can help decipher the underlying biological mechanisms. Therefore, there is a strong incentive to develop powerful statistical methods that are capable of detecting these potential drug-protein interactions effectively.

Various methods have been proposed to address the drug-target prediction problems *in silico*. One common method is to predict the drugs interacting with a single given protein based on the chemical structure similarity in a classic classification framework. Keiser *et al. *[2,3] proposed a method to predict targets of proteins based on the chemical similarity of their ligands. This kind of approach, however, does not take advantage of the information in the protein domain. Another widely-used method is molecular docking [4] which requires the non-trivial modeling of 3D structure of the target protein. Unfortunately the 3D structures of many proteins are not available [5], e.g., very few GPCRs have been crystallized.

Recently, some new approaches are proposed to perform drug-target prediction using both the chemical (drug chemical structure) and genomic (protein structure) spaces information [3,6,7]. In [6] the two spaces are encoded together by defining a pair wise kernel which is then fed to the support vector machine (SVM) for classification. The drawback of this kernel framework is that there will be a huge number of samples to be classified (i.e., number of drugs multiplies number of proteins) which poses significant computational complexity. Another problem is that the negative drug-protein pairs are selected randomly without experimental confirmation. Yamanishi *et al. *[7] developed a bipartite graph model where the chemical and genomic spaces as well as the drug-protein interaction network are integrated into a pharmacological space. In the bipartite model, the known interactions in the training data are labeled as +1 while all other unknown drug-protein pairs in the training data are assumed as non-interactions with label 0. Then three different classifiers are available: new drug candidate versus known target protein, known drugs versus new target protein and new drug candidate versus new target protein candidate. More recently, Bleakley and Yamanishi [8] proposed a state-of-the-art bipartite local model (BLM) by transforming edge-prediction problems into well-known binary classification problems. Nevertheless, the first flaw of the bipartite model, like the kernel SVM method [6], is that the unknown interactions of the drugs and proteins in the training data are all assumed non-interaction and cannot be inferred. We also prefer only one classifier to predict whether one drug-protein pair interacts or not. Lastly, all the methods did not utilize a wealth of unlabeled information to assist prediction.

In this paper, a semi-supervised learning method - Laplacian regularized least square (LapRLS) [9] is employed to utilize both the small amount of available labeled data and the abundant unlabeled data together in order to give the maximum generalization ability from the chemical and genomic spaces.

Further, the standard LapRLS is improved by incorporating a new kernel established from the known drug-protein interaction network (NetLapRLS). In our framework, the known interactions are labeled as +1 and all other unknown pairs are labeled as 0 to indicate they are going to be predicted. Two classifiers are trained on the drug and protein domains respectively and then are combined together to give the final prediction. Compared with a naive weighted profiled method, the proposed drug-protein interaction methods based on LapRLS and NetLapRLS obtain better results than using the labeled data alone. And the proposed NetLapRLS which incorporates drug-protein network information provides superior performance than standard LapRLS.

## Results and discussion

### Cross validation results analysis

The weighted profile method, standard LapRLS and NetLapRLS were evaluated on the four classes of target proteins including enzymes, ion channels, GPCRs and nuclear receptors. We carried out a ten-fold cross-validation by splitting the golden standard interaction dataset into 10 subsets. Each fold was then taken in turn as a test set and the remaining nine folds are used as training set. For example, there are 54 drugs and 26 proteins in the nuclear receptor data set with 90 known interactions. In each cross-validation, the 80 drug-protein pairs are used as the training data while the remaining 1,324 drug-protein pairs including the 10 positive interactions are designated as the testing data set. Thus the training sample is very small compared with the testing data set. This motivates us to employ the semi-supervised method that can utilize the information from the unlabeled samples to predict drug-protein interaction. The performance is evaluated using receiver operating curve (ROC) analysis [10]. For simplicity, we set *β*_*d *_= *β*_*p *_= 0.3, *γ*_*d*1 _= *γ*_*p*1 _= 1, and *γ*_*d*2 _= *γ*_*p*2 _= 0.01 for NetLapRLS. These parameters can be better selected by a further cross validation. If *γ*_*d*2 _and *γ*_*p*2 _are set to be 0, NetLapRLS becomes the standard LapRLS method. Table 1 shows the AUC (area under the ROC curve), sensitivity and specificity. The sensitivity and specificity are defined as TP/(TP+FN) and TN/(TN+FP), respectively. The cutoff for calculation of sensitivity and specificity is set to select the top pairs with the same number of the test set.

**Table 1:** Statistics of the prediction performance

Data	Methods	AUC	Sensitivity(%)	Specificity(%)
* Enzyme	Combining weighted profile	92.2	6	99.9
	LapRLS	95.0	53	99.9
	NetLapRLS	98.3	75	99.9
* Ion channel	Combining weighted profile	90.7	17	99.7
	LapRLS	96.1	36	99.8
	NetLapRLS	98.6	72	99.9
* GPCR	Combining weighted profile	86.9	13	99.7
	LapRLS	93.4	24	99.8
	NetLapRLS	97.1	50	99.8
Nuclear receptor	Combining weighted profile	81.0	11	99.4
	LapRLS	85.0	16	99.4
	NetLapRLS	88.8	21	99.5

The AUC is the area under the ROC curve, normalized to 100. The cutoff for sensitivity and specificity is set to select the number of the interactions in the test data.

**Figure 1:**
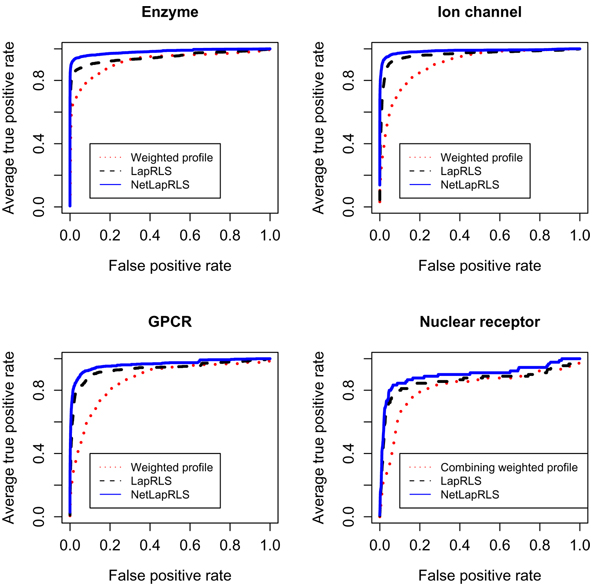
**ROC curves of cross validation**. ROC curves of the semi-supervised learning and the combining weighted profile methods for four classes target proteins: enzymes, ion channels, GPCRs and nuclear receptors.

From Table 1 and Figure 1, we can see that LapRLS and NetLapRLS methods, which use unlabeled information, provided better performance with respect to AUC score and sensitivity. Among the four data sets, the two semi-supervised learning methods provided the highest sensitivity scores in enzyme data set because there are most known interactions. The known interaction number is a key factor of our semi-supervised methods since the testing data set is much larger than training data set in our cross-validation setup. The proposed NetLapRLS which incorporates the drug-protein interaction network information obtained better result than the standard LapRLS, especially with respective to the sensitivity which is dramatically improved. On the four data sets, the sensitivity from NetLapRLS performed better than LapRLS by 42%, 100%, 108% and 31% respectively and, demonstrated the importance of network information. The improvement in sensitivity of NetLapRLS over LapRLS is most significant in ion channel data set because the inner-connection in the ion channel drug-protein interaction network is most complete according to the proportion of unreachable paths between drugs and proteins [7]. Yildirim *et al. *[11] concluded that there are an overabundance of 'follow-on' drugs from the topological analyses of current drug-protein network, that is, drugs that target already known proteins, i.e., me-too drugs. With the drug-protein network being completed fastly by high-throughput experimental and computational approaches, this network information is becoming critical in drug discovery.

### Comparison with bipartite local model [8]

Recently, Bleakley and Yamanishi [8] extended Yamanishi's bipartite method [12] to bipartite local model which is considered as state-of-the-art. The predictions from the drug domain and protein domain using SVM are combined together to form a final prediction by a maximum operation. We also employed this kind of integration by a mean operation. However, we used a semi-supervised learning method to handle the classification with small samples labeled which is difficult for traditional supervised classifiers. For instance, in the above cross-validation experiment of the nuclear receptor data set, the semi-supervised classifier is trained on 80 positive samples in order to make predictions on 1,324 unlabeled samples. In the BLM, the ten-fold cross-validation is performed on the drug and protein domains separately. The known interactions between the selected drugs and proteins are labeled as interaction while interactions between the drugs and proteins for training are regarded as non-interaction. Though we consider the undetermined relationship between drug-protein pair should not be labeled as non-interaction, we adopt the cross validation method in the BLM for the sake of comparison in the same condition. The comparison is performed in terms of AUC, area under precision-recall (AUPR), sensitivity, specificity and PPV as shown in Table 2. Sensitivity, specificity and PPV are calculated when the top one percentile in the prediction score is chosen as a cutoff because high-confidence prediction results are more useful in practical applications. We observed that BLM method outperformed our NetLapRLS in AUC and AUPR scores, but the performances of our NetLapRLS are comparable with BLM in sensitivity, specificity and PPV.

**Table 2:** Results of BLM and NetLapRLS based on cross validation experiments 5 times

Data	Methods	AUC	AUPR	Sensitivity(%)	Specificity(%)	PPV(%)
* Enzyme	BLM	96.8(0.1)	85.2(0.2)	83.2(0.2)	99.82(0.002)	82.3(0.2)
	NetLapRLS	95.6(0.3)	82.6(0.6)	81.0(0.5)	99.80(0.005)	80.2(0.5)
* Ion channel	BLM	97.2(0.1)	83.2(0.4)	28.0(0.03)	99.96(0.001)	96.4(0.1)
	NetLapRLS	94.7(0.3)	82.5(0.5)	28.4(0.14)	99.98(0.005)	98.1(0.5)
* GPCR	BLM	94.4(0.3)	65.0(1.6)	28.0(0.8)	99.83(0.02)	83.9(2.4)
	NetLapRLS	93.1(0.3)	66.0(1.5)	29.2(0.8)	99.87(0.03)	87.5(2.4)
Nuclear Receptor	BLM	84.1(0.9)	58.4(2.2)	14.0(0.6)	99.89(0.04)	90.0(3.9)
	NetLapRLS	85.6(1.8)	51.6(2.3)	15.1(1.0)	99.97(0.07)	97.1(6.1)

The AUC and AUPR scores are normalized to 100. The cutoff for sensitivity, specificity and PPV is set to choose the top one percentile in the predictoin score as positive.

Semi-supervised learning method is superior to the traditional supervised learning method when labeled samples are small along with large unlabeled samples available. In this cross validation setup, the unknown interactions are labeled as non-interaction in the training data set. So our NetLapRLS did not get good results in AUC and AUPR scores compared with BLM because most of samples are labeled. However, NetLapRLS still gave good prediction results in sensitivity, specificity and PPV. This indicated that NetLapRLS can provide a list of drug-protein interaction candidates with high confidence.

### Enzyme

**Figure 2:**
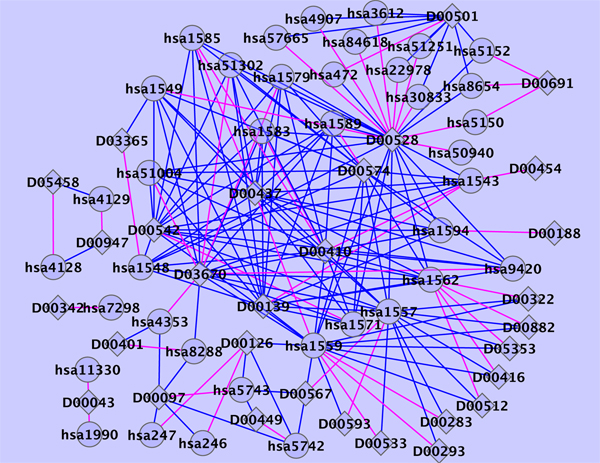
**Predicted enzyme interaction network**. Diamonds and circles represent drugs and target proteins, respectively. Blue and red lines indicate known interactions and newly predicted interactions with 50 highest scores, respectively.

Table 3 shows the list of the top 5 predicted drug-protein pairs, with annotation given in the KEGG database [13]. Searching the latest version of KEGG drug database and Drugbank [14], we found that the fifth highest scored drug-protein pair (D00097 and hsa5743) in Table 3 is annotated as an interaction. Figure 2 shows the predicted top 50 scoring drug-protein interaction network on the enzyme data using the all known interactions as the training data set.

**Table 3:** Top 5 scoring predicted drug-protein interactions for the enzyme data set

Rank	Pair	Annotation
*1	D00528	Anhydrous caffeine
	hsa1549	cytochrome P450, family 2, subfamily A, polypeptide 7
*2	D00542	Halothane
	hsa1571	cytochrome P450, family 2, subfamily E, polypeptide 1
*3	D00437	Nifedipine
	hsa1559	cytochrome P450, family 2, subfamily C, polypeptide 9
*4	D00410	Metyrapone
	hsa1585	cytochrome P450, family 11, subfamily B, polypeptide 2
*5	D00097	Salicylic acid
	hsa5743	prostaglandin-endoperoxide synthase 2

### Ion channel

**Figure 3:**
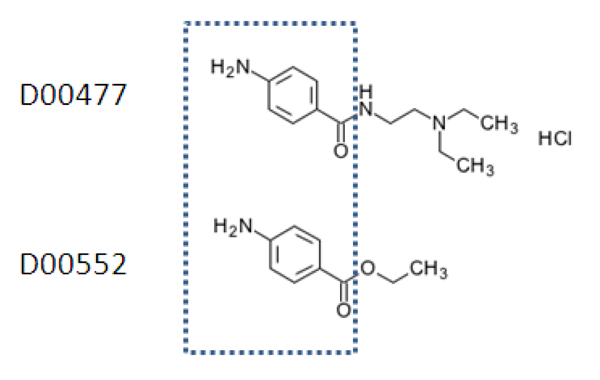
**Chemical structures**. Chemical structures of drug D00477 and D00552 (from KEGG).

**Figure 4:**
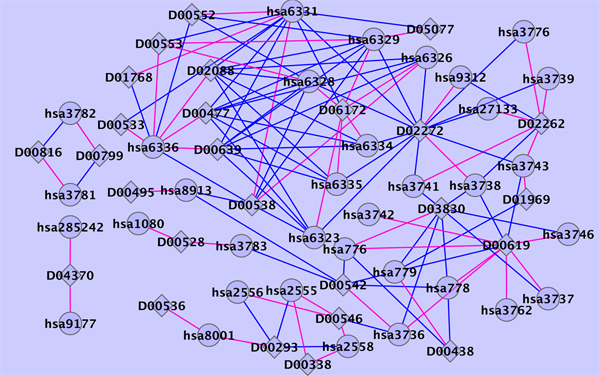
**Predicted ion channel interaction network**. Predicted ion channel interaction network. diamonds and circles represent drugs and target proteins, respectively. Bule and red lines indicate known interactions and newly predicted interactions with 50 highest scores, respectively.

Table 4 shows the list of the top five predicted drug-protein pairs on the ion channel data set, with annotation given in the KEGG database [13]. In the latest version of KEGG drug database, the targets of drug D00477 (rank 2 in table 4) include SCN1A, SCN2A, SCN3A, SCN4A, SCN5A, SCN8A and SCN9A. The targets of drug D00552 are SCN10A, SCN1A, SCN2A, SCN3A, SCN4A, SCN5A, SCN8A and SCN9A. Thus, our predicted target of D00552 is confirmed (rank 3 in Table 4). The targets of drugs D00477 and D00552 are very similar which can be explained by their common chemical structures in Figure 3. Based on the chemical structure similarity, we predict that SCN10A is also a target of drug D00477 (rank 2 in table 4), as the interaction between SCN10A and D00552 is known. Rank 5 in table 4 predicts GABAR2 is one of the targets of drug D00546. This prediction is reasonable because in Drugbank D00546 is annotated to interact with GABAR1 which is very similar with GABAR2 in sequence and function. Figure 4 shows the predicted top 50 scoring drug-protein interaction network on the ion channel data set using the all known interactions as training data set.

**Table 4:** Top 5 scoring predicted drug-protein interactions for the ion channel data set

Rank	Pairs	Annotation	
*1	D00438	Nimodipine	
	hsa779	calcium channel, voltage-dependent, L type, alpha 1 S subunit, beta 2	
*2	D00477	Procainamide hydrochloride	
	hsa6336	sodium channel, voltage-gated, type X, alpha subunit(SCN10A)	
*3	D00552	Ethyl aminobenzoate	
	hsa6331	sodium channel, voltage-gated, type V, alpha subunit(SCN5A)	
*4	D02272	Quinidine sulfate	
	hsa3738	potassium voltage-gated channel, shaker-related subfamily, member 3	
*5	D00546	Desflurane	
	hsa2555	gamma-aminobutyric acid (GABA) A receptor, alpha 2(GABAR2)	

#### GPCRs

**Figure 5:**
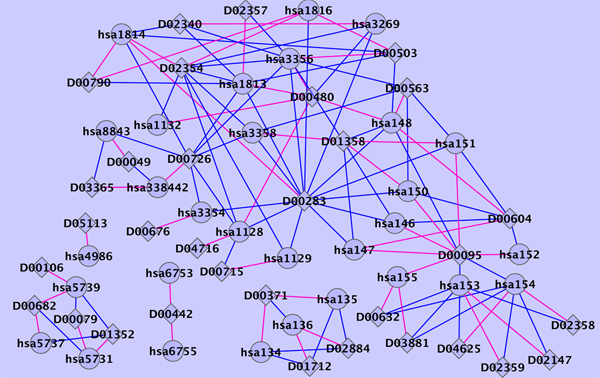
**Predicted GPCRs interaction network**. Predicted GPCRs interaction network. diamonds and circles represent drugs and target proteins, respectively. Blue and red lines indicate known interactions and newly predicted interactions with 50 highest scores, respectively.

Table 5 shows the list of the top five predicted drug-protein pairs on GPCRs data set, with annotation given in KEGG database. Based on the most recent KEGG database, the predictions of rank 2 and 3 in Table 5 are confirmed. Additionally, six predicted new targets (hsa146, hsa147, hsa150, hsa151, hsa152 and hsa155) of drug adrenaline (D00095) from the newly predicted interactions with 50 highest scores are also annotated as an interaction in the latest KEGG drug database. Ranks 4 and 5 in Table 5 predict both D02345 and D00283 target protein DRD3. In Drugbank, D02345 and D00283 are annotated to interact with protein DRD1, DRD2, and DRD4. Because DRD3 is very similar with those proteins in function, our method predicts DRD3 is also the target of drugs D02345 and D00283. This result demonstrated our method employed the information from protein domain. Figure 5 shows the predicted top 50 scoring drug-protein interaction network on the GPCRs data set using the all known interactions as the training data set.

**Table 5:** Top 5 scoring predicted drug-protein interactions for the GPCRs data set

Rank	Pair	Annotation	
*1	D02358	Metoprolol	
	hsa154	adrenergic receptor, beta 2	
*2	D00095	Adrenaline	
	hsa155	beta3-adrenergic receptor agonist	
*3	D00371	Theophylline	
	hsa135	adenosine A2a receptor antagonist	
*4	D02354	Thiethylperazine	
	hsa1814	dopamine receptor D3	
*5	D00283	Clozapine	
	hsa1814	dopamine receptor D3(DRD3)	

#### Nuclear receptor

**Figure 6:**
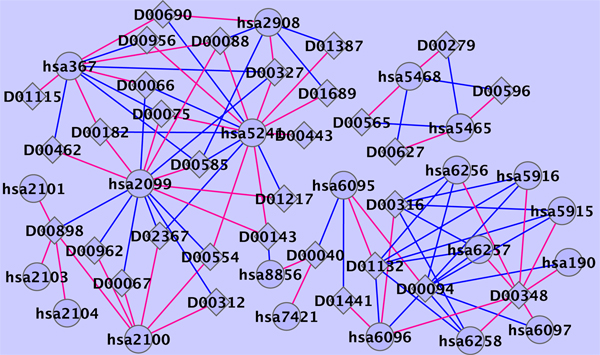
**Predicted nuclear receptor interaction network**. Predicted nuclear receptor interaction network. diamonds and circles represent drugs and target proteins, respectively. Blue and red lines indicate known interactions and newly predicted interactions with 50 highest scores, respectively.

Table 6 shows the list of the top 5 predicted drug-protein pairs on nuclear receptor data set, among which four predictions are about drug D00348. In Drugbank, drug D00348 is annotated to interact with protein (retinoic acid receptor, alpha). The two predicted targets with the highest scores (hsa5915 and hsa5916) of drug D00348 are both from retinoic acid receptor class. Those proteins are probably as the targets of the same protein due to their similarity in sequence and function. Figure 6 shows the predicted top 50 scoring drug-protein interaction network on the nuclear receptor data set with the all known interactions as the training data set.

**Table 6:** Top 5 scoring predicted drug-protein interactions for the nuclear receptor data set

Rank	Pair	Annotation	
*1	D00348	Isotretinoin	
	hsa5915	retinoic acid receptor, beta	
*2	D00348	Isotretinoin	
	hsa5916	retinoic acid receptor, gamma	
*3	D00182	Norethindrone	
	hsa2099	estrogen receptor 1	
*4	D00348	Isotretinoin	
	hsa6256	retinoid X receptor, alpha	
*5	D00348	Isotretinoin	
	hsa6257	retinoid X receptor, beta	

## Conclusions

In this work, we presented a semi-supervised learning method NetLapRLS for drug-protein interaction prediction by integrating information from chemical space, genomic space and drug-protein interaction network space. Our method has no use of the negative samples and predicts the interaction of each drug-protein pair. The results we obtained when predicting human drug-target interaction networks involving enzymes, ion channels, GPCRs, and nuclear receptors demonstrated the superior performance of NetLapRLS. Furthermore, recently added drug-target interactions to the KEGG immediately allowed us to confirm some strongly-predicted drug-target interactions on the four data sets obtained using our method. This enhances the strength of our proposed method for realistic drug-target prediction application.

The ideal way to use semi-supervised learning for predicting compound-protein interactions is to incorporate information from different biological spaces by a multi-task kernel and is fed to classical semi-supervised learning. However, the implementation of such a large scale semi-supervised learning method will be computationaly costly. Our future work, will incorporate more sophisticated and biologically relevant information into the kernel similarity, such as side effect [15], to improve the prediction accuracy.

## Methods

Semi-supervised learning (SSL) has been attracting much research attention in the machine learning community [16]. SSL provides better prediction accuracy by using unlabeled information. Here we employ a data-dependent manifold regularization framework which uses the geometry of the probability distribution [9]. One of the implementations of this framework is the Laplacian regularized least squares (LapRLS) which is simple and has comparable performance with Laplacian regularized support vector machine.

Consider the drug dataset $D=\{d_{1},\ldots ,d_{n_{d}}\}$ and the target protein dataset $\mathbb{P}=\{p_{1},\ldots ,p_{n_{p}}\}$ where *n_d _*and *n_p _*are the numbers of the drugs and proteins in the study respectively. An interaction pattern of drug *d_i _*and target protein *p_j _*is represented by a binary label matrix $Y\in {ℬ}^{n_{d}\times n_{p}}$. If drug *d_i _*is known to interact with target protein *p_j_*, **Y***_ij _*= 1 otherwise **Y***_ij _*= 0. Given the 'gold standard' drug-target interactions, the goal is to infer their unknown interactions. Two classifiers will be trained using LapRLS on the chemical and genomic spaces separately, followed by a combination of the two classifiers. A supervised learning method is suitable in this case. However the known interactions from public databases are still extremely small compared to the whole drug-target interaction space. Another issue is that we only have the information of the interactions, but do not know which drug target pair has no interaction, i.e., no negative samples in the training process. Herein we first test a simple supervised weighted profile method. Then the standard LapRLS and drug-protein interaction network incorporated NetLapRLS are extended to predict the drug-protein interaction.

### Materials

The data used here is downloaded from (http://web.kuicr.kyoto-u.ac.jp/supp/yoshi/drugtarget/) [7]. Here below we provide a brief description.

#### • Chemical data

The chemical structure similarity between compounds are calculated by SIMCOMP [17] using chemical structures fetched from KEGG LIGAND database. SIMCOMP provides a global similarity score by the ratio between the size of common substructures and the size of the union structures of two compounds. Applying this operation to all compounds pairs, we constructed a similarity matrix denoted $Y\in {ℛ}^{n_{d}\times n_{p}}$ which represents the chemical space information.

#### • Genomic data

A normalized Smith-Waterman score is calculated to indicate the similarity between two amino acid sequences of target proteins which were obtained from the KEGG GENES database. All protein pairs similarities are computed to construct a similarity matrix denoted $S_{p}\in {ℛ}^{n_{p}\times n_{p}}$ which represents the genomic space.

#### • Drug-protein interaction data

At the time of the paper [7] was written, Yamanishi *et al. *[7] found 445, 210, 223, and 54 drugs targeting 664 enzymes, 204 iron channels, 95 GPCRs, and 26 nuclear receptors, receptively, and the known interactions are 2926, 1476, 635 and 90.

### Combining weighted profiles

The method of combining weighted profiles follows the idea that the label of the new sample is determined by its similarity with the training samples. For a drug *d_i_*, its interaction *f*(*d_i_*, *p_j_*) with a protein *p_j _*in ℙ is predicted with the following formulation:

(1) $$f(d_{i},p_{j})=\frac{1}{N_{d_{i}}}\sum_{k=1}^{n_{d}}s_{d}(d_{i},d_{k})Y_{kj}$$

where *s_d_*(*d_i_*, *d_k_*) is a chemical structure similarity score from S*_d _*and ${\text{N}}_{d_{i}}$ is a normalization term defined as ${\text{N}}_{d}=\sum_{k=1}^{n_{d}}s_{d}(d_{i},d_{k})$. Meanwhile, for a protein *p_j _*, its interaction *f*(*p_j _*, *d_i_*) with a drug *d_i _*can also be calculated in the genomic space by:

(2) $$f(p_{j},d_{i})=\frac{1}{{\text{N}}_{p_{j}}}\sum_{k=1}^{n_{p}}s_{p}(p_{j},p_{k})Y_{ik}$$

where s*_p_*(*p_j _*, *p_k_*) is a genomic sequence similarity score from **S***_p _*and ${\text{N}}_{p_{i}}$ is a normalization term defined by ${\text{N}}_{p_{j}}=\sum_{k=1}^{n_{p}}s_{p}(p_{j},p_{k})$ Note that Equations (1) and (2) are estimating the interaction of the same drug-protein pair (*d_i _*~ *p_j_*) from different data sources. The two predictions should be combined to give the final prediction by

(3) $$\bar{f}(d_{i},p_{j})=\frac{f(d_{i},p_{j})+f(p_{j},d_{i})}{2}.$$

**Figure 7:**
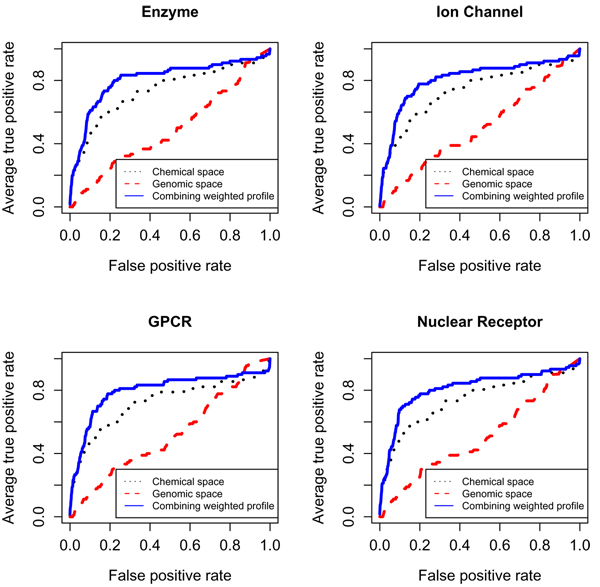
**The ROC curves of combining weighted profile**. The ROC curves of combining weighted profile, weighted profile from chemical and genomic spaces on GPCR data.

The drug-protein pairs (*d_i_*, *p_j_*) in $\bar{f}$(*d_i_*, *p_j_*) with high scores are predicted to interact each other. The original weighted profile method is used in [7]. However their predictions in the two spaces are not fused. Figure 7 shows the method of combining weighted profiles provides better prediction than methods using the single space on the four data sets.

### LapRLS and NetLapRLS for drug-protein interaction prediction

In LapRLS and NetLapRLS, the data-dependent regularization terms are normalized Laplacian operation on graphs. Herein two undirected graphs of drug domain and protein domain including both labeled and unlabeled samples are represented by $G_{d}=\{V_{d},{ℰ}_{d}\}$ and $G_{p}=\{V_{p},{ℰ}_{p}\}$, where the set of nodes or vertices is $V_{d}=\{d_{i}\},V_{p}=\{p_{i}\}$ and the set of edges is ℰ_*d *_= {*ed_mn_*}, ℰ_*p *_= {*ed_mn_*} respectively. Each drug *d_i _*or protein *p_j _*is treated as the node on the graph and the weight of edge *ed_mn_*{*ep_mn_*} is *wd_mn_*(*wp_mn_*).

Typically, the weight measures the similarity between two nodes. In our case, the drug domain similarity **W**_*d *_= {*wd*_*mn*_} is obtained by combining the chemical similarity **S**_*d *_and drug-target interaction network. The protein domain similarity **W***_p _*= {*wp*_*mn*_} is derived by combining the genomic similarity **S***_p _*and drug-protein interaction network spaces. The chemical similarity **S***_d _*and genomic similarity **S***_p _*have already been introduced in Section Materials.

**Figure 8:**
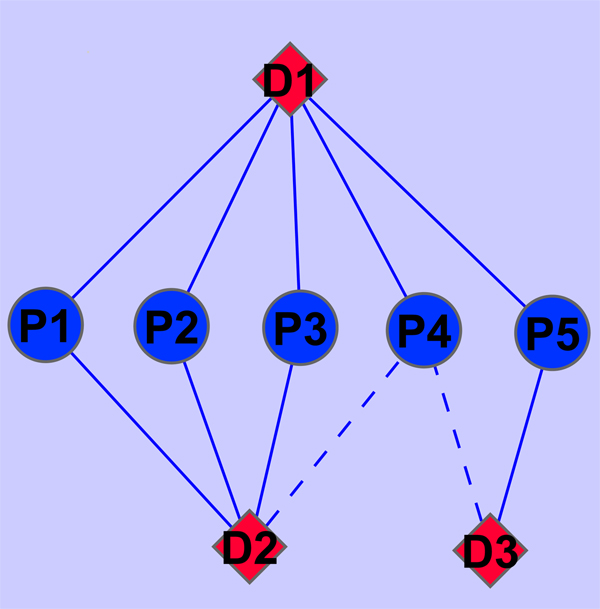
**The example of drug-protein interaction network**. The example of drug-protein interaction network.

Next we need to extract the information from the drug-protein interaction network space. The underlying assumption made here is that if two drugs share more target proteins, they are more similar. For example, in Figure 8, the blue line means the known drug-protein interaction while the red line represents the interaction to be predicted. So drug D2 shares 3 same proteins with drug D1 while drug D3 shares a common protein with drug D1. Drug D1 interacts with Protein P4. Based on the assumption here, we can infer that it is more probable that drug D2 interacts with protein P4 than drug D3 does. So another similarity matrix for drug domain from drug-protein interaction network $K_{d}\in {ℛ}^{n_{d}\times n_{d}}$ can be established whose each entry is the number of proteins shared by drug *d*_*i *_and *d*_*j*_. Similarly, we can also derive the network similarity matrix $K_{p}\in {ℛ}^{n_{p}\times n_{p}}$ whose each entry is the number of drugs shared by protein *p*_*j *_and *p*_*i*_. Though drug-protein interaction network was also used in [7], our method employs a different way to extract information from the network. The shortest path concept is used in [7] while we utilize the number of common nodes shared by two proteins(drugs) to indicate a new similarity measurement.

Now the drug domain similarity **W***_d _*can be derived from the chemical similarity and drug-protein network similarity by linear combination $W_{d}=\frac{\gamma_{d1}S_{d}+\gamma_{d2}K_{d}}{\gamma_{d1}+\gamma_{d2}}$. Similarly, the protein domain similarity **W***_p _*can be obtained by $W_{p}=\frac{\gamma_{p1}S_{p}+\gamma_{p2}K_{p}}{\gamma_{p1}+\gamma_{p2}}$. Compared with the standard LapRLS, our NetLapRLS incorporates drug-protein network information into the prediction model. In the following paragraph, we just describe the method NetLapRLS from which the standard LapRLS can be deduced by setting γ_M*d*2 _= γ_*p*2 _= 0.

Given the similarity matrices of drug domain and protein domain, we first perform Laplacian operation on the two graphs which is required by our semi-supervised learning method. The node degree matrices **D***_d _*and **D***_p _*are two diagonal matrices with their (*k*, *k*)-element defined as $D_{d}(k,k)=\sum_{m=1}^{n_{d}}wd_{k,m}$ and $D_{p}(k,k)=\sum_{m=1}^{n_{p}}wp_{k,m}$. The Laplacian operation of the two graphs is defined as Δ*_d _*= **D***_d _*- **W***_d _*and Δ*_p _*= **D***_p _*- **W***_p _*respectively. The normalized graph Laplacians are $L_{d}=D_{d}^{-1/2}\Delta_{d}D_{d}^{-1/2}=I_{n_{d}\times n_{d}}-D_{d}^{-1/2}W_{d}D_{d}^{-1/2}\;\text{and}\;L_{p}=D_{p}^{-1/2}\Delta_{p}D_{p}^{-1/2}=I_{n_{p}\times n_{p}}-D_{p}^{-1/2}W_{p}D_{p}^{-1/2}$ respectively.

NetLapRLS defines a continuous classification function **F **that is estimated on the graph to minimize a cost function. The cost function typically enforces a trade-o between the smoothness of the function on the graph of both labeled and unlabeled data and the accuracy of the function at fitting the label information for the labeled nodes. Herein we extend NetLapRLS to the matrix form. The two continuous classification functions are defined by $F_{d}\in {ℛ}^{n_{d}\times n_{p}}$ and $F_{p}\in {ℛ}^{n_{p}\times n_{d}}$. Let's first address the prediction **F***_d _*on the drug domain. The cost function of NetLapRLS is defined as follows

(4) $$F_{d}^{*}=\underset{F_{d}}{\min}J(F_{d})=||Y-F_{d}|{|}_{ℱ}^{2}+\beta_{d}Trace(F_{d}^{T}L_{d}F_{d})$$

where $\|\cdot {\|}_{ℱ}$ is Frobenius norm and *Trace *is the trace of a matrix. Representer theorem [18] shows that the solution is a linear combination

$$F_{d}^{*}=W_{d}\alpha_{d}^{*}$$

Substituting this form into equation (4), we arrive at a convex differentiable objective function with respect to variable $\alpha_{d}\in {ℛ}^{n_{d}\times n_{p}}$

(5) $$\alpha_{d}^{*}=\arg\underset{\alpha_{d}\in {ℛ}^{n_{d}\times n_{p}}}{\min}\{||Y-W_{d}\alpha_{d}|{|}_{ℱ}^{2}+\beta_{d}Trace(\alpha_{d}^{T}W_{d}L_{d}W_{d}\alpha_{d})\}$$

The derivative of the objective function vanishes at the minimizer:

(6) $$-W_{d}(Y-W_{d}\alpha_{d})+\beta_{d}W_{d}L_{d}K_{d}\alpha_{d}=0$$

which leads to the following solution:

(7) $$\alpha_{d}^{*}={\left(W_{d}+\beta_{d}L_{d}W_{d}\right)}^{-1}Y$$

Then we get the prediction from the drug domain in the following form:

(8) $$F_{d}^{*}=W_{d}{\left(W_{d}+\beta_{d}L_{d}W_{d}\right)}^{-1}Y$$

Similarly, we can also derive the prediction in the protein domain by

(9) $$F_{p}^{*}=W_{p}{\left(W_{p}+\beta_{p}L_{p}W_{p}\right)}^{-1}Y^{T}$$

In the end, the predictions from drug and protein domains are combined into

(10) $$F^{*}=\frac{F_{d}^{*}+{\left(F_{p}^{*}\right)}^{T}}{2}$$

## Competing interests

The authors declare that they have no competing interests.

## Authors' contributions

Zheng Xia and Ling-Yun Wu co-developed the method, implemented the code and drafted the manuscript. Xiaobo Zhou and Stephen T.C. Wong supervised this project and gave critical revision of the manuscript. Stephen Wong provided the financial support for the work from his bioinformatics and bioengineering program grant from The Methodist Hospital Research Institute (TMHRI), Houston, Texas. All authors read and approved the manuscript.
